# Uso de inteligencia artificial en la predisposición genética a enfermedad crítica por COVID-19: evaluación comparativa de modelos de aprendizaje automático

**DOI:** 10.1515/almed-2024-0129

**Published:** 2025-04-02

**Authors:** Salomon Martin Perez, Flora Sanchez Jimenez, Sandra Fuentes Cantero, Marta Jímenez Barragan, Catalina Sanchez Mora, Juan M. Borreguero Leon, Teresa Arrobas Velilla, Agustín Valido Morales, Juan A. Delgado Torralbo, Antonio León-Justel

**Affiliations:** Unidad de Bioquímica clínica, Hospital Universitario Virgen Macarena, Sevilla, España; Servicio Análisis clínicos, Hospital General Rio Tinto, Huelva, España; Unidad de Neumología, Hospital Universitario Virgen Macarena, Sevilla, España

**Keywords:** aprendizaje automático, COVID-19, enfermedad crítica, inteligencia artificial, polimorfismos genéticos (SNPs), regresión logística

## Abstract

**Objetivos:**

La predicción temprana de enfermedad crítica por COVID-19 es crucial para optimizar el manejo clínico. Este estudio tiene como objetivo optimizar la predicción de enfermedad crítica por COVID-19 mediante la integración de datos clínicos, de laboratorio y polimorfismos genéticos en modelos de inteligencia artificial, evaluando y comparando el rendimiento de distintos algoritmos de aprendizaje automático.

**Métodos:**

Se analizaron 155 pacientes hospitalizados, 23 de los cuales desarrollaron enfermedad crítica. Se realizó un análisis univariante para evaluar la asociación entre siete SNPs y 9 variables clínicas y 10 parámetros de laboratorio en la analítica al ingreso.

**Resultados:**

De los 7 SNPS, solo tres SNPs se asociaron significativamente con enfermedad crítica: rs77534576, rs10774671 y rs10490770. Los modelos de ensemble consiguieron el mejor rendimiento: Random Forest (AUC=0,989), XGBoost (AUC=0,954) y AdaBoost (AUC=0,927). La importancia de las variables varió entre los modelos, destacando la edad, proteína C reactiva, cardiopatías y los tres SNPs en la mayoría de ellos. La incorporación de los SNPs mejoró el poder predictivo en comparación con estudios previos sin datos genéticos. La validación interna confirmó la superioridad y estabilidad de los modelos de ensemble.

**Conclusiones:**

Los modelos de aprendizaje automático pueden ayudar en la predicción por enfermedad crítica por Covid-19. La incorporación de SNPs asociados a gravedad a los datos clínicos y de laboratorio mejora el poder predictivo. Se requieren estudios adicionales con cohortes más grandes y diversas para validar y generalizar estos hallazgos antes de su aplicación clínica.

## Introducción

La pandemia de COVID-19 ha tenido un impacto profundo y duradero en la medicina a nivel mundial, desafiando la capacidad de adaptación de los sistemas de salud y poniendo de manifiesto carencias que habían permanecido inadvertidas. Uno de los grandes desafíos es analizar la gran gran variabilidad en la gravedad de la COVID-19 entre los pacientes, desde casos leves o asintomáticos hasta individuos que desarrollan una enfermedad crítica por COVID-19 [1] El riesgo de mortalidad está determinado por una combinación de factores, incluyendo la vulnerabilidad a la infección viral y la propensión a desarrollar una inflamación pulmonar [2]. Cabe destacar que la gravedad ha cambiado significativamente según la cepa viral predominante y otros factores, entre los cuales la inmunización de la población ha jugado un papel crucial [3].

La inteligencia artificial nos ofrece nuevas oportunidades y herramientas, desempeñando un papel clave en la pandemia de COVID-19, con aplicaciones en diagnóstico, seguimiento, rastreo, desarrollo de fármacos y vacunas, y reducción de la carga asistencial, facilitando la monitorización de la crisis y la investigación [4]. La crisis sanitaria global fomentó la colaboración científica global, generando rápidamente datos clave del SARS-CoV-2, incluyendo genomas de referencia [5] y factores genéticos de susceptibilidad. Diversos proyectos internacionales, como estudios GWAS y de exoma completo [6], 7] han investigado la variabilidad interpersonal frente al virus, y se han determinado una serie de polimorfismos (SNPs) que se asociarían una susceptibilidad genética a una mayor gravedad [8], [9], [10].

Con el objetivo de optimizar la predicción de enfermedad crítica por COVID-19, se propone el desarrollo de modelos de aprendizaje automático, incorporando los SNPs asociados a gravedad, así como datos clínicos y de laboratorio haciendo un estudio comparativo entre ellos. Primero se analizará la asociación de los SNPs candidatos con la enfermedad y solo aquellos significativamente asociados se incorporarán a los modelos. Además se evaluará la importancia de las variables asociadas en cada modelo con el fin de determinar el papel de los SNPs en cada predicción y estudiar la influencia de estos polimorfismos en la predicción.

## Materiales y métodos

En el presente estudio se incluyeron pacientes que acudieron al servicio de Urgencias del Hospital Universitario Virgen Macarena de Sevilla mayores de 18 años y tuvieron diagnóstico confirmado de COVID-19 mediante RT-PCR (reacción en cadena de la polimerasa con transcriptasa inversa) en el dispositivo Xpert^®^ Xpress SARS-CoV-2 de la empresa Cepheid entre mayo de 2020 y enero de 2021. Las muestras fueron recolectadas por el Biobanco del hospital con el consentimiento específico de los pacientes para el uso de su material genético y proporcionadas al laboratorio para el análisis de los SNPs. Este estudio cuenta con la aprobación del comité de ética e investigación clínica.

La variable de estudio definida como enfermedad crítica por COVID-19 se refiere a la ocurrencia de al menos uno de los siguientes eventos durante la hospitalización: ingreso en la unidad de cuidados intensivos (UCI), necesidad de ventilación invasiva o fallecimiento. Esta definición, fundamentada en investigaciones previas sobre resultados graves de COVID-19, se utilizó como una variable categórica (sí/no) para nuestro resultado principal [11], 12]. Esta información se extrajo de la revisión de la historia clínica electrónica de cada paciente.

Para la creación de los modelos se usaron datos clínicos, datos de laboratorio y datos de SNPs. Los datos clínicos se extrajeron de los registros médicos electrónicos, incluyendo variables dicotómicas (sexo, hallazgos radiológicos pulmonares, que se definen como imágenes sugerentes de alteración pulmonar en radiografía/TC, cardiopatías, hipertensión, diabetes, enfermedades autoinmunes, incluyendo lupus, artritis reumatoide, psoriasis y miastenia gravis, tabaquismo e Infecciones respiratorias previas, registradas en el mes anterior al ingreso y una variable continua (edad). Las variables analíticas continuas, obtenidas de la primera determinación al ingreso, comprenden: proteína C reactiva, creatina quinasa, creatinina, D-dímero, recuento de linfocitos, alanina aminotransferasa, plaquetas, urea, hemoglobina y lactato.

Se realizó un análisis de datos faltantes en las variables de laboratorio al ingreso y se imputaron los valores ausentes utilizando la mediana de cada variable antes de los análisis estadísticos.

Los SNPs incluidos fueron: rs10490770, ubicado cerca de los genes LZTFL1 y LOC107986083 en el cromosoma 3 [13], 14]; rs10774671, en el gen OAS1 del cromosoma 12; rs77534576, entre los genes del cromosoma 17 [13], 14]; rs2109069, en el gen DPP9 del cromosoma 19 [15], 16]; rs74956615, cerca de los genes FDX2 y RAVER1 en el cromosoma 19 [17]; y rs2834158, en el gen IFNAR2 del cromosoma 21 [6], 18], todos ellos incluidos en el estudio PreMed-Covid19 [19]. A esta selección, nuestro añadimos el SNP rs35705950, localizado en el gen MUC5B del cromosoma 11 [20].

El análisis de los polimorfismos se llevó a cabo una vez recolectadas todas las muestras, las cuales fueron entregadas congeladas al laboratorio. Se realizó inicialmente la extracción de ADN a partir de sangre periférica. El estudio de las variantes genéticas se llevo a cabo en el analizador Cobas Z 480 (Roche Diagnostics GmbH) mediante PCR en tiempo real. Se evaluó la asociación entre polimorfismos y la enfermedad crítica, analizando cada uno de forma individual usando regresión logística. Se consideraron cuatro modelos posibles de herencia genética: dominante, recesivo, aditivo y codominante. Se verificó el cumplimiento del equilibrio de Hardy-Weinberg mediante una prueba de chi-cuadrado antes del análisis de regresión logística. Los genotipos se codificaron según cada modelo de herencia. Para cada SNP el modelo más adecuado se determinó comparando el ajuste del modelo codominante con los otros modelos, usando la prueba de razón de verosimilitud y el criterio de información de Akaike. Finalmente se estableció un umbral de p<0,20 para incluir SNPs en modelos predictivos.

Todos los modelos utilizaron la totalidad de las variables disponibles. En los modelos de aprendizaje automático, la selección de variables se realizó automática. Sin embargo, en el modelo de regresión logística, la selección de variables se basó en criterios estadísticos.

### Modelo de regresión logística

Se comenzó con una transformación de las variables numéricas en binarias (edad, hemoglobina, plaquetas, linfocitos, dímeros, urea, creatinina, lactato deshidrogenasa, alanina transaminasa, creatina quinasa y proteína C reactiva), para facilitar la interpretación clínica y la aplicabilidad práctica, permitiendo establecer puntos de corte claros para la toma de decisiones, utilizando análisis basados en curvas ROC y el estadístico de Youden para establecer los puntos de corte óptimos. Se evaluó la multicolinealidad entre las variables predictoras utilizando el Factor de Inflación de la Varianza (VIF). Las variables con un VIF mayor a 5 fueron excluidas. Se realizo un análisis univariante para evaluar la asociación individual de cada variable predictora con la variable objetivo. Se ajustaron modelos de regresión logística univariantes para cada variable. Se incluyeron en el modelo de regresión logística multivariable aquellas variables con una prevalencia de al menos 5 % y aquellas con un valor p bilateral <0,20 en el análisis univariante.

### Modelos de aprendizaje automático

En el preprocesamiento de datos, las variables numéricas continuas se normalizaron utilizando la clase StandardScaler de scikit-learn, que implementa la normalización Z-score. En el preprocesamiento se dividió el conjunto de datos en 80 % para entrenamiento y 20 % para prueba y se aplicó SMOTE para equilibrar las clases al conjunto de datos de entrenamiento. Para evaluar la robustez y estabilidad de los modelos, se realizaron dos validaciones internas complementarias: una validación cruzada de 5 pliegues (k-fold) y una validación bootstrap con 1,000 iteraciones para evaluar la robustez y estabilidad mediante remuestreo con reemplazo.

El análisis estadístico se realizó con Python 3.8, utilizando pandas 1.2.4 para el manejo de datos, scikit-learn 0.24.2 para los modelos predictivos y métricas de evaluación, imbalanced-learn 0.8.0 para el balanceo de clases mediante SMOTE, xgboost 1.4.2 para el modelo XGBoost, statsmodels 0.12.2 para el análisis estadístico y matplotlib 3.4.2 para la visualización de resultados.

Se evaluaron seis modelos, aportando cada uno estrategias diferentes: KNN clasifica según la similitud con datos vecinos; Random Forest combina árboles de decisión para mejorar precisión y reducir sobreajuste; AdaBoost ajusta pesos de instancias mal clasificadas; XGBoost destaca por su alto rendimiento con árboles potenciados por gradiente; SVM con kernel de base radial busca el hiperplano óptimo para separar clases; y Naive Bayes utiliza el teorema de Bayes asumiendo independencia entre características. Se usó GridSearchCV en la cohorte de entrenamiento para optimizar los hiperparámetros.

### Evaluación de modelos

El rendimiento de cada modelo se evaluó utilizando varias métricas, siendo la principal el área bajo la curva ROC (AUC), que mide la capacidad del modelo para distinguir entre las diferentes clases. Las métricas adicionales incluyeron exactitud (proporción de predicciones correctas), precisión (verdaderos positivos entre predicciones positivas), sensibilidad (verdaderos positivos entre casos realmente positivos), y el puntaje F1 (media armónica de precisión y sensibilidad). También se analizó la importancia de cada variable en cada modelo, mediante un método de permutación que mide el impacto en el rendimiento del modelo al alterar aleatoriamente cada variable. Los valores se normalizaron a porcentajes para permitir comparaciones entre modelos. Para la regresión logística, se utilizaron los coeficientes del modelo para cuantificar la influencia de cada variable predictora en el resultado.

## Resultados

La cohorte del estudio incluyó un total de 155 pacientes hospitalizados, de los cuales 23 evolucionaron hacia una enfermedad crítica. Las variables analizadas se clasificaron en dos categorías: numéricas y dicotómicas. Se encontraron datos faltantes en las siguientes variables de laboratorio: creatina quinasa (CK) con 12 valores (7,79 %), dímeros D y lactato deshidrogenasa (LDH) con 4 valores cada uno (2,60 %), y proteína C reactiva (PCR) con 1 valor (0,65 %) que fueron imputados por la mediana de los datos.

En la Tabla 1 se presentan las variables dicotómicas analizadas. Los alelos rs77534576, rs10490770 y rs10774671 fueron más frecuentes en pacientes críticos, con porcentajes de 17,4 %, 34,8 % y 21,7 % respectivamente, en comparación con 4,5 %, 18,9 % y 9,8 % en los no críticos. Además, la hipertensión y la enfermedad cardiaca fueron más comunes en el grupo crítico, con un 60,9 % y 39,1 % respectivamente, frente a un 36,4 % y 18,2 % en los no críticos. El 73,9 % de los pacientes críticos ingresaron en planta, frente al 81,8 % de los no críticos, ya que muchos críticos fueron directamente a cuidados intensivos. Otras variables como la infección, enfermedad autoinmune, diabetes, ser fumador, y hallazgos radiológicos no mostraron diferencias tan marcadas entre los grupos.

**Tabla 1:** Distribución de variables dicotómicas.

Variable dicotómica	General (n=155)	Enf. crítica (n=23)	Sin enf. crítica (n=132)
rs77534576	10 (6,5 %)	4 (17,4 %)	6 (4,5 %)
rs10490770	33 (21,3 %)	8 (34,8 %)	25 (18,9 %)
rs10774671	18 (11,6 %)	5 (21,7 %)	13 (9,8 %)
Infección	4 (2,6 %)	1 (4,3 %)	3 (2,3 %)
Enfermedad autoinmune	6 (3,9 %)	0 (0 %)	6 (4,5 %)
Hipertensión	62 (40 %)	14 (60,9 %)	48 (36,4 %)
Diabetes	26 (16,8 %)	4 (17,4 %)	22 (16,7 %)
Enfermedad cardiaca	33 (21,3 %)	9 (39,1 %)	24 (18,2 %)
Fumador	19 (12,3 %)	3 (13 %)	16 (12,1 %)
Ingreso en planta	125 (80,6 %)	17 (73,9 %)	108 (81,8 %)
Hallazgos radiológicos	115 (74,2 %)	18 (78,3 %)	97 (73,5 %)

La Tabla muestra el conteo y porcentaje de pacientes con características específicas en tres grupos: el grupo general (n=155), pacientes con enfermedad (Enf.) crítica (n=23), y pacientes sin enfermedad crítica (n=132). Los porcentajes se calculan en función del total de cada grupo.

La Tabla 2 presenta las variables numéricas analizadas. La edad mediana fue mayor en pacientes críticos (69 vs. 58 años). Los niveles de hemoglobina fueron similares (14,6 g/dL) en ambos grupos, con valores mínimos más bajos en críticos (12,7 vs. 13,5 g/dL). El conteo de plaquetas fue ligeramente superior en los críticos (239 vs. 221 × 10^3^/µL). Los niveles de dímeros y creatinina también fueron más altos en pacientes críticos (585 vs. 486 ng/mL y 1 vs. 0,9 mg/dL, respectivamente). La lactato deshidrogenasa mostró valores ligeramente mayores en los críticos (296 vs. 275 U/L), mientras que la creatina quinasa fue notablemente más elevada en este grupo (111,5 vs. 75,5 U/L). Los niveles de proteína C reactiva fueron significativamente más altos en pacientes críticos (79 vs. 50,7 mg/L). Los niveles de urea y linfocitos mostraron valores similares entre grupos. Se mantuvieron en el análisis ya que en nuestro estudio previo [11] estas variables presentaron diferencias más marcadas entre los grupos.

**Tabla 2:** Distribución de variables numéricas.

Variable númerica	General (n=155)	Enf. crítica (n=23)	Sin enf. crítica (n=132)
Edad, años	59,5 [46–69]	69 [62–73,5]	58 [45–66]
Hemoglobina, g/dL	14,6 [13,4–15,4]	14,6 [12,7–15,4]	14,6 [13,5–15,4]
Plaquetas, × 10^3^/µL	222 [173–285,8]	239 [137,5–268]	221 [175,5–287]
Linfocitos, × 10^3^/µL	1,3 [0,9–1,6]	1,3 [1–1,8]	1,2 [0,9–1,6]
Dimeros, ng/mL	488 [337,2–835,2]	585 [390–977,5]	486 [335–776]
Urea, mg/dL	32 [25–41]	33 [28–45]	31 [25–41]
Creatinina, mg/dL	0,9 [0,8–1,1]	1 [0,8–1,2]	0,9 [0,7–1,1]
Lactato deshidrogenasa, U/L	276 [209,2–342]	296 [200–361,5]	275 [211–338,5]
Alanina transaminasa, U/L	31 [20–50]	27 [20,5–34,5]	32 [20–51,5]
Creatina quinasa, U/L	79 [50–138]	111,5 [58,8–213]	75,5 [48–126,2]
Proteina C reactiva, mg/L	55,8 [25,5–102,2]	79 [57,2–133,9]	50,7 [22,2–96,9]

La Tabla presenta la mediana y el rango intercuartílico (Q3-Q1) de diversas variables numéricas en tres grupos de pacientes: el grupo general (n=155), pacientes con enfermedad (Enf.) crítica (n=23), y pacientes sin enfermedad crítica (n=132).

### Análisis de polimorfismos

Solo tres SNPs fueron seleccionados para incluirlos en los modelos predictivos. Esta selección se basó en un análisis exhaustivo que incluyó el equilibrio de HW y la comparación de modelos de herencia.

El análisis del equilibrio de HW, mostrado en la Tabla Suplementaria 1, reveló que seis de los siete polimorfismos estudiados cumplen con este equilibrio tanto en casos como en controles. Estos son: rs2834158, rs35705950, rs74956615, rs2109069, rs77534576 y rs10490770. Sus valores de chi-cuadrado oscilan entre 0,01 y 2,46, con p-valores superiores a 0,05, lo que indica que no hay diferencias significativas entre las frecuencias alélicas esperadas y las observadas. Sin embargo, el polimorfismo rs10774671 mostró una desviación significativa del equilibrio de HW en el grupo de casos (χ^2^=7,99, p=0.005), mientras que se mantuvo en equilibrio en el grupo de controles (χ^2^=1,86, p=0,173).

Tras comparar cada modelo de herencia con el modelo codominante (Tabla Suplementaria 2), se identificaron tres SNPs que cumplen con el criterio de p<0,20 para su inclusión en los modelos predictivos. Estos son: rs77534576, para el cual se seleccionó un modelo de herencia aditivo; rs10774671, que mostró un mejor ajuste con un modelo codominante, y rs10490770, también con un modelo aditivo.

### Modelos

**Figura 1:**
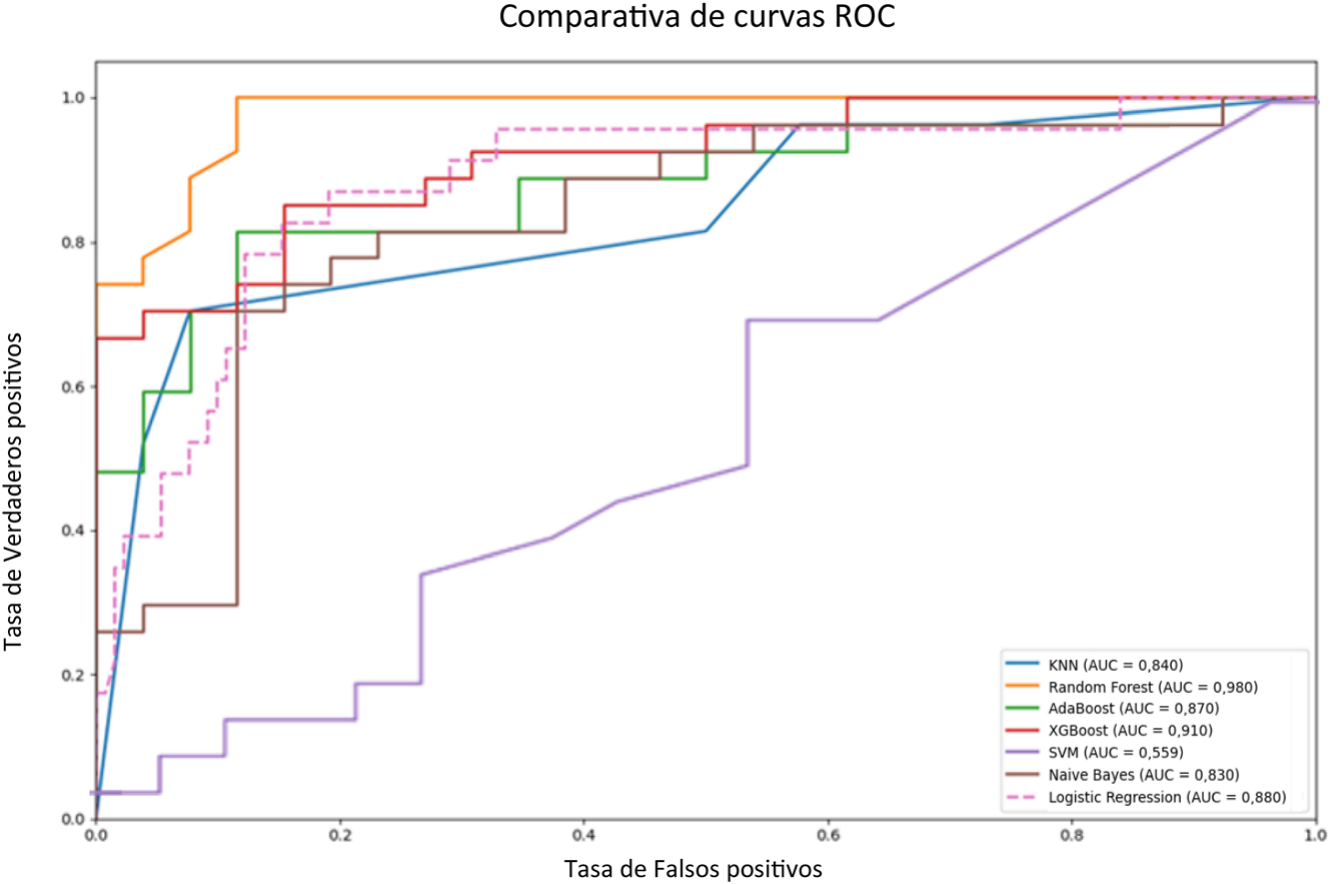
Curvas ROC comparativas de los diferentes modelos. Curvas ROC comparativas de diferentes modelos de aprendizaje automático. La gráfica muestra la tasa de verdaderos positivos frente a la tasa de falsos positivos para cada modelo. El área bajo la curva (AUC) es una medida de la capacidad del modelo para distinguir entre clases, en este caso, enfermedad crítica por COVID-19.

Los modelos de aprendizaje automático superaron en general a la regresión logística en la predicción de enfermedad crítica por COVID-19. En la Figura 1 se muestran las curvas ROC de cada modelo, donde podemos apreciar que los modelos de ensamble, como Random Forest (AUC=0,98), AdaBoost (AUC=0,87) y XGBoost (AUC=0,91), tendieron a mostrar un rendimiento superior en términos de AUC comparados con los modelos individuales. Estos últimos incluyen KNN (AUC=0,84), SVM (AUC=0,36), Naive Bayes (AUC=0,83) y Regresión Logística (AUC=0.88).

La Tabla 3 muestra todas las métricas, siendo el área bajo la curva ROC (AUC) la medida principal de evaluación. Random Forest se destacó como el modelo más eficaz entre todos los algoritmos, logrando el AUC más alto (0,989), también obtuvo la mayor exactitud (90,6 %), la segunda mejor precisión (92,3 %), una alta sensibilidad (88,9 %) y el F1-score más elevado (0,906), superando significativamente a la regresión logística tradicional en todas las métricas. XGBoost logró el segundo mejor AUC con 0,954 y la precisión más alta, alcanzando un 95,2 %. Sin embargo, su sensibilidad fue del 74,1 %, inferior a la de Random Forest. Su exactitud fue del 84,9 % y su F1-score de 0,833, ambos los segundos mejores después de Random Forest. Por otro lado, AdaBoost obtuvo el tercer mejor AUC con 0,927, presentando una exactitud del 81,1 %, una precisión del 87,0 %, una sensibilidad del 74,1 % y un F1-score de 0,800.

**Tabla 3:** Métricas de modelos.

Modelo	Exactitud	Precisión	Sensibilidad	F1-score	AUC	VP	FP	FN	VN	Total
Random Forest	0,906	0,923	0,889	0,906	0,989	24	2	3	24	53
XGBoost	0,849	0,952	0,741	0,833	0,954	20	1	7	25	53
AdaBoost	0,811	0,870	0,741	0,800	0,927	20	3	7	23	53
Logistic Regression	0,877	0,750	0,261	0,387	0,881	6	2	17	28	53
Naive Bayes	0,735	0,685	0,888	0,774	0,830	24	11	3	15	53
KNN	0,679	0,639	0,852	0,730	0,823	23	13	4	13	53
SVM	0,528	0,538	0,519	0,528	0,559	14	12	13	14	53

Métricas para varios modelos de clasificación evaluados en términos de exactitud, precisión, sensibilidad, F1-score, área bajo la curva (AUC) y valores de la matriz de confusión (VP, FP, FN, VN). Los modelos evaluados son KNN, Random Forest, AdaBoost, XGBoost, SVM, Naive Bayes y Regresión Logística.

El modelo de regresión logística tras aplicar VIF incluyó las siguientes variables: edad (≥65 años), los marcadores genéticos rs10774671, rs10490770 y rs77534576, presencia de cardiopatías, hipertensión arterial, y los parámetros analíticos: recuento de linfocitos (≥1,94 × 10^3^/µL), niveles de creatina quinasa (≥102 U/L), proteína C reactiva (≥62,50 mg/L), dímeros D (≥942 ng/mL), creatinina (≥1,06 mg/dL), lactato deshidrogenasa (≥296 U/L) y urea (≥27 mg/dL). A pesar de lograr la segunda mejor exactitud (87,7 %), obtuvo un AUC de 0,881, inferior a los tres modelos de ensamble mencionados. Además, presentó la sensibilidad más baja (26,1 %) y el F1-score más bajo (0,387) entre todos los modelos, aunque su precisión (75 %) fue moderada.

Los modelos restantes mostraron resultados variados. Naive Bayes alcanzó un AUC de 0,830, con la segunda mejor sensibilidad (88,8 %) después de SVM, pero con una precisión más baja (68,5 %). KNN obtuvo un AUC de 0,823, con una sensibilidad alta (85,2 %) pero la segunda precisión más baja (63,9 %). SVM, a pesar de tener la sensibilidad más alta (92,5 %), mostró el AUC más bajo (0,559) y la exactitud más baja (50,9 %), lo que sugiere un posible sobreajuste a la clase positiva.

El análisis de la importancia de las variables para los modelos de aprendizaje automático (Tabla 4) y el modelo de regresión logística (Tabla 5, Figura Suplementaria 1) reveló diferencias interesantes entre los modelos. Random Forest identificó el polimorfismo rs10774671 (14,14 %), las plaquetas (12,12 %), y los polimorfismos rs77534576 (10,10 %) y rs10490770 (7,07 %) como las variables más importantes. XGBoost destacó la presencia de cardiopatías (37,25 %), la creatina quinasa (11,76 %) y la proteína C reactiva (10,46 %) como los predictores más relevantes. AdaBoost priorizó la proteína C reactiva (14,59 %), los dímeros D (11,35 %) y el hallazgo radiológico (10,81 %). KNeighbors dio mayor importancia a los dímeros D (54,10 %), las plaquetas (17,21 %) y la alanina aminotransferasa (9,84 %). SVM mostró una fuerte preferencia por los dímeros D (81,82 %), seguidos por el lactato deshidrogenasa (13,64 %). Naive Bayes consideró la edad (9,14 %), la hipertensión y la alanina aminotransferasa (8,60 % cada una) como los factores más influyentes. Finalmente, la regresión logística identificó la edad ≥65 años (13,59 %), el polimorfismo rs10774671 (12,80 %) y los linfocitos ≥1,94 (10,65 %) como los factores más importantes.

**Tabla 4:** Porcentaje de importancia de variables por modelo de IA.

Variable	Random Forest, %	KNeighbors, %	AdaBoost, %	XGBoost, %	SVM, %	Naive Bayes, %
Edad	6,06	0,00	1,62	7,19	9,57	9,14
Proteína C reactiva	3,03	6,56	14,59	10,46	17,38	1,08
Cardiopatías	7,07	0,00	1,62	37,25	0,35	5,38
Hipertensión	1,01	0,00	2,70	9,15	12,77	8,60
Creatina quinasa	5,05	1,64	2,16	11,76	4,26	2,15
rs10774671	14,14	0,00	2,16	3,92	12,41	5,91
Creatinina	8,08	0,00	7,03	0,00	0,00	4,84
Dímeros D	0,00	54,10	11,35	5,23	0,00	4,84
Linfocitos	2,02	0,00	1,08	0,00	0,71	6,45
Alanina aminotransferasa	6,06	9,84	10,27	0,00	0,00	8,60
Plaquetas	12,12	17,21	5,41	0,00	2,48	0,00
Urea	0,00	0,82	9,19	0,00	3,55	2,69
Hemoglobina	0,00	0,00	0,00	2,61	603	1,08
rs10490770	7,07	0,00	5,41	3,27	1,77	1,61
Lactato deshidrogenasa	2,02	9,84	1,08	0,65	4,26	2,15
Diabetes	10,10	0,00	0,54	0,00	0,00	5,38
rs77534576	10,10	0,00	3,24	1,31	10,99	6,99
Sexo (hombre)	1,01	0,00	5,41	2,61	1,77	5,91
Radiológico	0,00	0,00	10,81	0,00	0,00	2,69
Planta	4,04	0,00	3,24	4,58	1,77	0,00
Fumador	1,01	0,00	1,08	0,00	0,00	0,54
Infección	0,00	0,00	0,00	0,00	13,12	8,06
Autoinmune	0,00	0,00	0,00	0,00	2,13	5,91

Esta Tabla muestra la importancia relativa de cada variable en los modelos Random Forest, K-Neighbors, AdaBoost, XGBoost, SVM y Naive Bayes.

**Tabla 5:** Porcentaje de importancia de variables en el modelo de regresión logística.

Variable	Coeficiente	Importancia, %	Odds Ratio	Valor p
Edad (≥65)	1,16	13,59	5,81	<0,001
Gen (rs77534576)	1,10	12,80	4,39	0,04
Linfocitos (≥1,94)	0,91	10,65	2,54	0,10
Creatina quinasa (≥102)	0,90	10,56	2,85	0,03
Proteína C reactiva (≥62,50)	0,84	9,77	3,92	0,01
Gen (rs10774671)	0,75	8,71	2,52	0,15
Gen (rs10490770)	0,66	7,69	2,05	0,14
Cardiopatías	0,59	6,93	2,87	0,05
Hipertensión	0,55	6,38	2,69	0,04
Dímeros D (≥942)	0,34	3,95	2,73	0,05
Creatinina (≥1,06)	0,31	3,60	2,42	0,08
Lactato deshidrogenasa (≥296)	0,27	3,20	1,89	0,17
Urea (≥27)	0,19	2,18	2,32	0,19

Esta Tabla muestra la importancia relativa de cada variable en el modelo de regresión logística. Las variables numéricas incluyen el punto de corte establecido. La columna Coeficiente representa la magnitud y dirección de la asociación entre la variable y el resultado, la columna Importancia (%) indica la contribución relativa de cada variable en el modelo, el Odds Ratio muestra la razón de probabilidades entre casos y controles, y el Valor p indica la significación estadística de la asociación. Se selección un punto de corte de p<0,20, para su inclusión en el modelo.

Los SNPs mostraron una importancia variable según el modelo. El polimorfismo rs10774671 fue especialmente importante para Random Forest (14,14 %) y la regresión logística (12,80 %). El rs77534576 destacó en Random Forest (10,10 %) y tuvo una importancia moderada en la regresión logística (7,68 %). Por su parte, el rs10490770 mostró una importancia moderada tanto en Random Forest (7,07 %) como en la regresión logística (8,71 %).

### Validación interna

La validación interna mediante bootstrapping con 1,000 iteraciones reveló mejoras generalizadas en comparación con los resultados originales (Tabla Suplementaria 3). El modelo de Random Forest mantuvo su posición de liderazgo, con una exactitud de 95,6 % ± 3,0 % y AUC de 0,994 ± 0,008, seguido de cerca por los modelos XGBoost y AdaBoost, que mostraron mejoras significativas respecto a la modelo inicial (94,4 % ± 3,6 % y 93,2 % ± 3,8 % respectivamente). KNN también experimentó un aumento sustancial (80,6 % ± 5,8 %), mientras que la Regresión Logística mantuvo un rendimiento similar pero con alta variabilidad en precisión y sensibilidad. Naive Bayes mostró estabilidad, y SVM, aunque mejoró ligeramente, reveló un rendimiento inestable. En general, los modelos de ensemble demostraron superioridad y mayor estabilidad.

## Discusión

El principal hallazgo de este estudio es que los modelos de inteligencia artificial mejoran el poder predictivo de enfermedad critica por COVID-19 respecto al modelo de regresión logística clásica. Particularmente los modelos de tipo ensemble fueron superiores, obteniendo mejor rendimiento el modelo de Random Forest con un AUC de 0.989, seguido de XGBoost con 0,954 y de AdaBoost con 0,927 respecto a la Regresión Logística con 0,881. Los modelos han revelado patrones distintivos en la predicción de COVID-19 crítico, donde cada algoritmo enfatiza diferentes aspectos predictivos. En Random Forest, valores elevados de plaquetas y la presencia de los polimorfismos rs10774671, rs77534576 y rs10490770 incrementan el riesgo de enfermedad crítica, con pesos de importancia del 12,12 %, 14,14 %, 10,10 % y 7,07 % respectivamente. XGBoost señala que la presencia de cardiopatías es el predictor más fuerte (37,25 %), seguido por niveles elevados de creatina quinasa (11,76 %) y proteína C reactiva (10,46 %). AdaBoost identifica que mayores niveles de proteína C reactiva (14,59 %) y dímeros D (11,35 %) orientan hacia un mayor riesgo. La consistencia en la identificación de estos factores a través de múltiples modelos, especialmente los SNPs y marcadores inflamatorios, refuerza su validez como predictores robustos de COVID-19 crítico, aunque su peso relativo varía según el algoritmo empleado.

En la evaluación de la predisposición genética, solo tres SNPs de los siete se asociaron con enfermedad critica por COVID-19 en nuestra cohorte de pacientes (rs77534576, rs10774671 y rs10490770). Todos los modelos a excepción de SVM y KNN, identificaron los SNPs como factores significativos, si bien su importancia relativa varió entre ellos.

Los modelos de ensemble, como Random Forest, AdaBoost y XGBoost, demostraron un rendimiento sobresaliente, reforzando su idoneidad para este tipo de tarea predictiva al capturar patrones sutiles cruciales para identificar pacientes en riesgo. Sorprendentemente, el modelo KNN, pese a su simplicidad, logró un desempeño respetable. Por otro lado, la Regresión Logística, aunque presentó una alta exactitud global, mostró una baja sensibilidad. Sin embargo, su interpretabilidad sigue siendo una ventaja significativa en entornos médicos.

La incorporación de SNPs asociados a gravedad mejora la predicción de enfermedad crítica por COVID-19. En un estudio previo de nuestro grupo [11] sobre enfermedad crítica por COVID-19 sin incorporar los SNPs, el modelo de regresión logística mostró una AUC de 0,850. En contraste, el modelo de regresión actual, que incluye los SNPs, ha mejorado a una AUC de 0,881. Estas mejoras sugieren que la inclusión de datos genéticos ha incrementado la capacidad predictiva del modelo.

El análisis del equilibrio de Hardy-Weinberg mostró que, con la excepción de rs10774671 en el grupo de casos, todos los SNPs cumplieron con el equilibrio tanto en casos como en controles, lo que respalda la validez de nuestros hallazgos genéticos. De los siete SNPs analizados, tres tenían significancia estadística con la enfermedad crítica por COVID-19: rs77534576, rs10774671 y rs10490770. Para rs77534576 y rs10490770, se identificó un modelo de herencia aditivo como el más apropiado, sugiriendo un efecto acumulativo de cada alelo variante en el riesgo de enfermedad grave. Esto es consistente con los hallazgos de Yi Lin et al. [21], donde el rs77534576 fue uno de los SNPs asociado a hospitalizaciones y síntomas respiratorios muy severos debido al COVID-19. Para rs10774671, nuestro análisis favoreció un modelo codominante, indicando efectos distintos para cada genotipo. Este resultado se alinea con estudios previos, como el de El Yousfi et al. [13] y Huffman et al. [14], que han destacado el papel protector del alelo G de rs10774671 contra la enfermedad grave por COVID-19. Observamos una desviación del equilibrio de Hardy-Weinberg para rs10774671, habiéndose identificado una menor proporción relativa de individuos con el alelo protector en nuestra población de casos con respecto a los controles. Respecto a rs10490770, nuestros hallazgos respaldan su relevancia en la predicción de la gravedad de COVID-19, en línea con el estudio de Nakanishi et al. [22], aunque contrastan con los resultados no significativos reportados por Prajjval P et al. [23] en población india. Estas discrepancias subrayan la importancia de considerar la diversidad genética entre poblaciones en la interpretación de los resultados. Respecto al rs35705950, no fue significativo en contraste con. Van Moorsel et al. que demostraron que el alelo T de MUC5B rs35705950 confiere protección contra COVID-19 grave [20].

En el análisis comparativo sobre la importancia de cada variable destacamos que variables como la edad, los niveles de Proteína C Reactiva, la existencia de cardiopatías y los tres SNPs (rs10490770, rs10774671 y rs77534576) aparecen como relevantes en todos los modelos a excepción de SVM y KNN, aunque su importancia relativa varía. La diversidad en la importancia de las variables entre los modelos subraya la complejidad del problema y sugiere que un enfoque que combine múltiples modelos podría proporcionar una comprensión más robusta de los factores predictivos. La identificación de estos marcadores genéticos no solo mejora nuestra comprensión de los mecanismos subyacentes a la susceptibilidad al COVID-19 grave, sino que también abre nuevas vías para la medicina personalizada en el manejo de la pandemia.

La integración de estos factores genéticos en modelos predictivos, junto con variables clínicas y de laboratorio, representa un avance significativo hacia una estratificación del riesgo más precisa y personalizada. Sin embargo, es crucial reconocer que la predisposición genética es solo una pieza del rompecabezas, y su interpretación debe considerarse en el contexto más amplio de factores ambientales, comorbilidades y la respuesta inmune individual. Las diferencias en la importancia de las variables entre los modelos subrayan la necesidad de considerar múltiples enfoques para obtener una comprensión más completa del problema.

El estudio presenta limitaciones como el reducido tamaño de la muestra de 155 pacientes, lo que puede llevar a sobreajuste en modelos complejos, esto ocurre debido a la desproporción entre el número de observaciones y predictores del modelo, lo que provoca que éste se ajuste al ruido específico de la muestra en lugar de capturar las verdaderas relaciones poblacionales. Además, no se especificó si los valores analíticos iniciales correspondían al momento del ingreso en Urgencias o en UCI, lo que podría influir en la interpretación de los resultados debido a la evolución temporal de los biomarcadores. Aunque se usaron técnicas como la validación cruzada para mitigar estos problemas, es esencial validar tanto interna como externamente los hallazgos en cohortes más grandes y diversas antes de su aplicación clínica. Además, la limitada bibliografía sobre metodologías específicas para asociar SNPs con variables concretas o establecer el tipo de modelo de dominancia representa un desafío metodológico común en estudios de asociación genética.

Nuestro estudio destaca la superioridad de los algoritmos de aprendizaje automático, especialmente los modelos de tipo ensemble, en la predicción de enfermedad crítica por COVID-19. La incorporación de SNPs a las variables clínicas y analíticas mejora el poder predictivo. De los siete SNPs analizados, tres mostraron significancia estadística con la enfermedad crítica por COVID-19: rs77534576, rs10774671 y rs10490770, integrándose en los algoritmos y sugiriendo una predisposición genética a sufrir enfermedad crítica por COVID-19. Se recomienda la realización de estudios adicionales con cohortes más grandes y diversas para validar y extrapolar estos resultados, incluyendo una validación externa en poblaciones independientes.

## Supplementary Material

Supplementary Material

Supplementary Material

Supplementary Material

Supplementary Material

## Material Suplementario

Este artículo incluye material suplementario (https://doi.org/10.1515/almed-2024-0129).
