# Supplementary material for: Uso de inteligencia artificial en la predisposición genética a enfermedad crítica por COVID-19: evaluación comparativa de modelos de aprendizaje automático
Source: Adv Lab Med. 2025 Apr 2;6(2):190–8. [Article in Spanish] doi: 10.1515/almed-2024-0129 (PMC12107414; doi:10.1515/almed-2024-0129)
Supplement: Supplementary file 1 — Supplementary Material [file j_almed-2024-0129_suppl_001.docx]

**Tabla suplementaria 1**. Métricas de equilibrio de HW para casos y controles.

|  | **CASOS** | | | **CONTROLES** | | |
| --- | --- | --- | --- | --- | --- | --- |
| **Polimorfismo** | **CHI2** | **P-VALUE** | **Equilibrio HW** | **CHI2** | **P-VALUE** | **Equilibrio HW** |
| **rs2834158** | 1,308 | 0,253 | SI | 0,469 | 0,494 | SI |
| **rs35705950** | 2,457 | 0,117 | SI | 0,011 | 0,917 | SI |
| **rs74956615** | 0,048 | 0,827 | SI | 0,357 | 0,550 | SI |
| **rs2109069** | 1,431 | 0,232 | SI | 0,083 | 0,773 | SI |
| **rs77534576** | 0,198 | 0,656 | SI | 0,072 | 0,789 | SI |
| **rs10774671** | 7,987 | 0,005 | NO | 1,855 | 0,173 | SI |
| **rs10490770** | 0,195 | 0,659 | SI | 0,668 | 0,414 | SI |

Se muestra los resultados del valor de p y Chi2 para cada uno de los SNPs estudiados y si cumple el equilibrio de HW para los casos y controles.
