# Supplementary material for: Uso de inteligencia artificial en la predisposición genética a enfermedad crítica por COVID-19: evaluación comparativa de modelos de aprendizaje automático
Source: Adv Lab Med. 2025 Apr 2;6(2):190–8. [Article in Spanish] doi: 10.1515/almed-2024-0129 (PMC12107414; doi:10.1515/almed-2024-0129)
Supplement: Supplementary file 2 — Supplementary Material [file j_almed-2024-0129_suppl_002.docx]

**Tabla suplementaria 2**. Análisis tipos de dominancia para los SNPs estudiados

| **SNP** | **modelo** | **P_value** | **AIC** | **LRT_P_Value** | **Mejor ajuste** | **P < 0,20** |
| --- | --- | --- | --- | --- | --- | --- |
| **rs2834158** | **Codominante** | 0,407 | 134,048 | - | Codominante | No |
|  | **Dominante** | 0,473 | 133,333 | 0,257 |  |  |
|  | **Recesivo** | 0,390 | 133,108 | 0,303 |  |  |
|  | **Aditivo** | 0,851 | 133,812 | 0,184 |  |  |
| **rs35705950** | **Codominante** | 0,464 | 134,313 | - | Recesivo | No |
|  | **Dominante** | 0,985 | 133,847 | 0,216 |  |  |
|  | **Recesivo** | 0,240 | 132,467 | 0,695 |  |  |
|  | **Aditivo** | 0,721 | 133,724 | 0,235 |  |  |
| **rs74956615** | **Codominante** | 0,853 | 133,812 | - | Codominante | No |
|  | **Dominante** | 0,853 | 133,812 | 1,000 |  |  |
|  | **Recesivo** | - | 131,847 | 0,853 |  |  |
|  | **Aditivo** | 0,855 | 133,812 | 1,000 |  |  |
| **rs2109069** | **Codominante** | 0,620 | 134,889 | - | Codominante | No |
|  | **Dominante** | 0,586 | 133,551 | 0,416 |  |  |
|  | **Recesivo** | 0,577 | 133,535 | 0,422 |  |  |
|  | **Aditivo** | 0,880 | 133,824 | 0,334 |  |  |
| **rs77534576** | **Codominante** | 0,051 | 133,805 | - | Aditivo | Si |
|  | **Dominante** | 0,051 | 133,805 | 1,000 |  |  |
|  | **Recesivo** | - | 135,614 | 0,051 |  |  |
|  | **Aditivo** | 0,038 | 133,805 | 1,000 |  |  |
| **rs10774671** | **Codominante** | 0,117 | 131,561 | - | Codominante | Si |
|  | **Dominante** | 0,597 | 133,568 | 0,045 |  |  |
|  | **Recesivo** | 0,132 | 131,577 | 0,156 |  |  |
|  | **Aditivo** | 0,706 | 133,707 | 0,042 |  |  |
| **rs10490770** | **Codominante** | 0,358 | 133,790 | - | Aditivo | Si |
|  | **Dominante** | 0,170 | 131,963 | 0,677 |  |  |
|  | **Recesivo** | 0,421 | 133,200 | 0,235 |  |  |
|  | **Aditivo** | 0,136 | 131,794 | 0,950 |  |  |
