# Supplementary material for: Uso de inteligencia artificial en la predisposición genética a enfermedad crítica por COVID-19: evaluación comparativa de modelos de aprendizaje automático
Source: Adv Lab Med. 2025 Apr 2;6(2):190–8. [Article in Spanish] doi: 10.1515/almed-2024-0129 (PMC12107414; doi:10.1515/almed-2024-0129)
Supplement: Supplementary file 3 — Supplementary Material [file j_almed-2024-0129_suppl_003.docx]

**Tabla suplementaria 3.** Métricas de validación interna.

| **Modelo** | **Exactitud** | **Precisión** | **Sensibilidad** | **F1-Score** | **AUC** |
| --- | --- | --- | --- | --- | --- |
| **KNN** | 0,806 ± 0,058 | 0,756 ± 0,082 | 0,905 ± 0,068 | 0,820 ± 0,059 | 0,904 ± 0,041 |
| **Random Forest** | 0,956 ± 0,030 | 0,956 ± 0,042 | 0,958 ± 0,045 | 0,956 ± 0,031 | 0,994 ± 0,008 |
| **AdaBoost** | 0,932 ± 0,038 | 0,926 ± 0,057 | 0,939 ± 0,052 | 0,931 ± 0,040 | 0,964 ± 0,032 |
| **XGBoost** | 0,944 ± 0,036 | 0,940 ± 0,053 | 0,948 ± 0,052 | 0,943 ± 0,038 | 0,986 ± 0,016 |
| **SVM** | 0,562 ± 0,095 | 0,585 ± 0,206 | 0,568 ± 0,321 | 0,514 ± 0,203 | 0,619 ± 0,183 |
| **Naive Bayes** | 0,726 ± 0,068 | 0,693 ± 0,095 | 0,825 ± 0,116 | 0,745 ± 0,073 | 0,824 ± 0,062 |
| **Logistic Regression** | 0,868 ± 0,060 | 0,535 ± 0,435 | 0,248 ± 0,238 | 0,312 ± 0,266 | 0,837 ± 0,11 |

Métricas de validación interna para varios modelos de clasificación. Se presentan las medias y las desviaciones estándar (±) de cinco métricas : exactitud, precisión, sensibilidad, F1-Score y área bajo la curva (AUC). Los modelos evaluados son KNN, Random Forest, AdaBoost, XGBoost, SVM, Naive Bayes y Regresión Logística
