# Supplementary material for: Uso de inteligencia artificial en la predisposición genética a enfermedad crítica por COVID-19: evaluación comparativa de modelos de aprendizaje automático
Source: Adv Lab Med. 2025 Apr 2;6(2):190–8. [Article in Spanish] doi: 10.1515/almed-2024-0129 (PMC12107414; doi:10.1515/almed-2024-0129)
Supplement: Supplementary file 4 — Supplementary Material [file j_almed-2024-0129_suppl_004.docx]

**Figura suplementaria 1.** Porcentaje de Importancia de Variables para el modelo de Regresión logística.


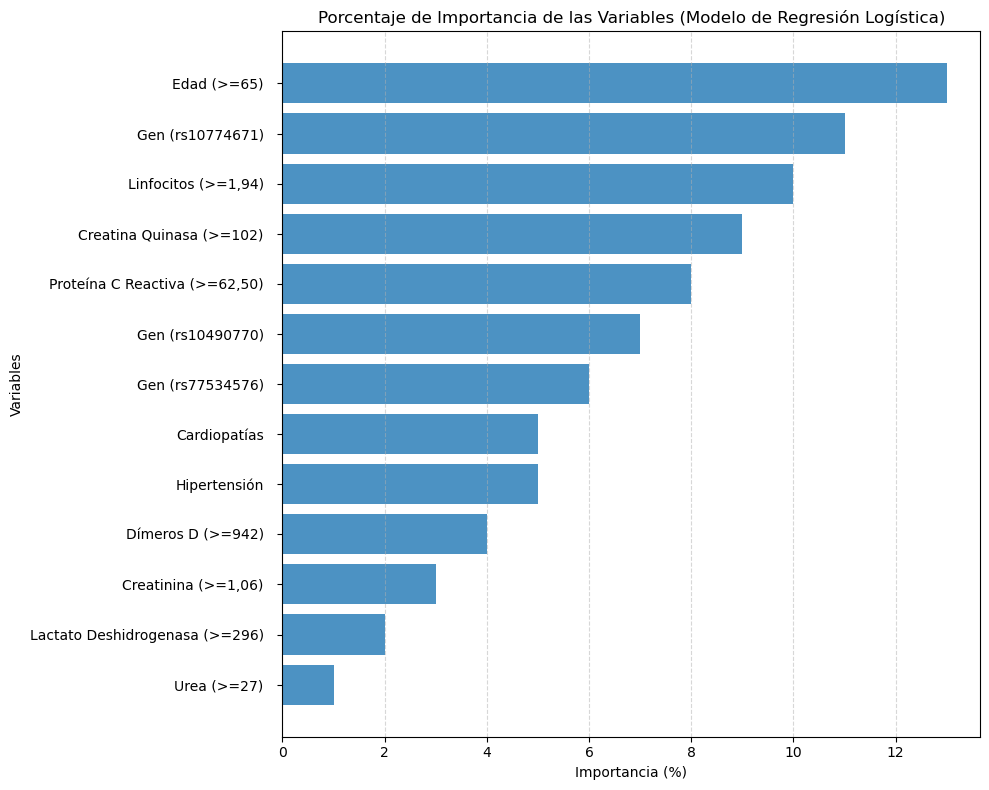


Este gráfico muestra la importancia relativa de cada variable en el modelo de regresión logística, la variables numéricas muestra el punto de corte seleccionado para cada una de ellas.
